# Identifying signals of memory from observations of animal movements

**DOI:** 10.1186/s40462-024-00510-9

**Published:** 2024-11-18

**Authors:** Dongmin Kim, Peter R. Thompson, David W. Wolfson, Jerod A. Merkle, L. G. R. Oliveira-Santos, James D. Forester, Tal Avgar, Mark A. Lewis, John Fieberg

**Affiliations:** 1 Department of Ecology, Evolution and Behavior, University of Minnesota, St. Paul, MN USA; 2 Department of Organismic and Evolutionary Biology, Harvard University, Cambridge, MA USA; 3 School of Environmental Science, Simon Fraser University, Burnaby, BC Canada; 4 Department of Fisheries, Wildlife and Conservation Biology, University of Minnesota, St. Paul, MN USA; 5 Department of Zoology and Physiology, University of Wyoming, Laramie, WY USA; 6 Departmento de Ecologia, Universidade Federal do Mato Grosso do Sul, Campo Grande, Brazil; 7 Department of Biology, University of British Columbia—Okanagan and Wildlife Science Centre, Biodiversity Pathways Ltd, Kelowna, BC Canada; 8 Department of Mathematics and Statistics and Department of Biology, University of Victoria, Victoria, BC Canada

**Keywords:** Animal movement, Cognitive map, Familiarity, Memory, Occurrence distribution, Space use, Step-selection analyses, Wildlife tracking data

## Abstract

Incorporating memory (i.e., some notion of familiarity or experience with the landscape) into models of animal movement is a rising challenge in the field of movement ecology. The recent proliferation of new methods offers new opportunities to understand how memory influences movement. However, there are no clear guidelines for practitioners wishing to parameterize the effects of memory on moving animals. We review approaches for incorporating memory into step-selection analyses (SSAs), a frequently used movement modeling framework. Memory-informed SSAs can be constructed by including spatial-temporal covariates (or maps) that define some aspect of familiarity (e.g., whether, how often, or how long ago the animal visited different spatial locations) derived from long-term telemetry data. We demonstrate how various familiarity covariates can be included in SSAs using a series of coded examples in which we fit models to wildlife tracking data from a wide range of taxa. We discuss how these different approaches can be used to address questions related to whether and how animals use information from past experiences to inform their future movements. We also highlight challenges and decisions that the user must make when applying these methods to their tracking data. By reviewing different approaches and providing code templates for their implementation, we hope to inspire practitioners to investigate further the importance of memory in animal movements using wildlife tracking data.

**Supplementary Information:**

The online version contains supplementary material available at 10.1186/s40462-024-00510-9.

## Introduction

Animal movement impacts ecological processes at all levels, including individual foraging efficiencies [16, 108], population persistence [24, 69], species distributions [38, 63], connectivity [26] and ecosystem functioning [12, 72, 99]. While much research has explored how animal movements are influenced by environmental conditions [38], intra- and inter-specific social interactions [63], and internal states such as hunger levels [49], the importance of past experience and memory is also increasingly recognized as a key component of animal movement [32, 60, 80]. For example, by remembering the location and outcomes of previously visited locations, many species can increase energy intake rates [50, 105] and lifespan [112], and avoid areas that might increase mortality risk [17, 43, 47]. Further, by remembering average environmental conditions, such as the average timing of resource waves, animals can better time migratory movements [2, 18]. Thus, models that incorporate memory are important for both developing and testing ecological theory, and they are likely to lead to improved predictions of how animals will respond to changes in their environment [33, 44].

One straightforward approach to integrating such complex types of memories into a model is to assume that past experiences can be encoded into a spatially-referenced system in the animal’s brain (sometimes referred to as a “cognitive map”), which is then accessed during the retrieval phase to inform movements. Although hidden from direct observation, a spatially referenced map can be mathematically modeled as a surface changing dynamically over time as memories are lost, reinforced, or replaced. These constructs are central to key empirical models for memory, including “time since last visit” to a location [90] as a determinant of wolf movement [91], and episodic returns of brown bears to ephemeral seasonal resources [102, 103]. Overall, the map is a latent, spatially-referenced variable, whose dynamics are inferred indirectly from animal movement patterns. Although the existence of cognitive maps in animals remains a subject of ongoing debate among researchers [6, 111], we use the term “cognitive map” here to broadly describe neurological or psychological mechanisms that allow animals to store and process spatial information. This definition is commonly used in much of the movement ecology literature [32, 60].

The information that animals gather, and perhaps memorize, as they move can be divided into three categories: (1) spatial information (i.e., locations animals have visited), (2) site attributes, including resource quality or quantity, and (3) temporal information (i.e., about how long-ago animals visited a previous site or when a site peaks in forage quality) [32, 55, 60, 102, 103, 106]. Movement ecologists often distinguish between ‘spatial memory’ that encodes spatial configurations (#1 from the description above) and ‘attribute memory’, which describes the attributes of local features (#2 and #3 from the description above) [32]. Food-caching blue jays (*Cyanocitta cristata*) use all three kinds of information: they remember the locations of many caches, the type of seed in each cache, and how long it has been since making the cache [23]. Owing to the temporal variability present in most environments, it can be advantageous to rely more heavily on recent experiences and to discount memories from long ago [25, 101]. For example, roe deer (*Capreolus capreolus*) primarily base their foraging decisions on recent experiences due to rapid changes in resource availability within their home ranges [85]. Note, however, that memory is also expected to temporally decay due to the limitations of the neurological infrastructure that holds it, and distinguishing such decay from an adaptive discounting may be particularly challenging [9]. By revisiting sites, animals can update their knowledge of site attributes, and optimal return times may depend on how quickly the reliability of past information decays due to environmental change as well as resource renewal rates [85, 98]. For example, wolves (*Canis lupus*) and brown bears (*Ursus arctos*) delay returning to previously visited kill sites so that prey numbers may recover [45, 92].

Ecologists have developed theoretical models to explore how past experiences and memory might influence animal movements [32, 39, 55, 60, 110]. The simplest encodable memory attribute is familiarity with a given location, either whether an individual has ever visited the site [75, 86] or how frequently it has visited the site in the past [17]. When coupled to spatial movement models, preference for familiar locations is sufficient for the formation of stable home ranges [42, 47, 66, 71, 84, 108]. More complex memory attributes include locations of resources or past conflicts, allowing animals to integrate spatial and attribute memory (i.e., memory of where positive and negative experiences occurred). Attraction to previously discovered resources can lead to resource-driven patterns of nonterritorial spatial segregation [1, 87]. By way of contrast, memory and avoidance of locations where past conflicts with conspecifics occurred can give rise to spontaneous territorial pattern formation [40, 82]).

Much of our understanding of the role of memory has come from ethologists and cognitive scientists studying animal behavior [53, 54, 95]. Early studies relied on observational data from experimental settings and found that food-storing birds frequently revisited sites to store and retrieve their resources (cache sites) [58, 59]. These behaviors may reflect the use of memory, but they may also be explained by birds preferring to store and revisit sites with certain characteristics [57]. To address this potential issue (which is still debated today, [79]), later ethologists used experimentation to separate out the role of memory-based and preference-based navigation by testing whether birds revisit their cache sites under different conditions (e.g., with and without food present) [94]. In addition, Balda and Kamil [11] forced food-caching Clark’s nutcrakers (*Nucifraga columbiana*) to store food at non-prefered locations and found these locations were still revisited.

Recent advances in animal tracking technology and statistical modeling approaches have motivated ecologists to explore the potential for memory-informed movements in a wide range of animal taxa (although terrestrial mammals, mainly ungulates, remain by far the most-studied group; see Table 1). By tracking individual animals over consecutive years, ecologists can identify whether current movements can be explained from observations of previously visited locations. These studies face challenges similar to those faced by early ethologists, namely that animals may revisit sites primarily because those sites have characteristics the animals prefer [79]. To overcome this inferential challenge, ecologists fit statistical models that include covariates capturing habitat preferences along with covariates reflecting familiarity [65, 67]. This approach attempts to use statistical controls rather than experimental controls to infer whether animals revisit sites more frequently than expected based on site characteristics alone. However, we must be cautious about attributing revisits to memory, even after adjusting for known habitat preferences, because we will rarely know all the environmental features that influence animal movements [60]. Animals may be responding to unmeasured environmental cues that we do not include in our model.

**Table 1:** Empirical modeling for memory

Paper	Species	Memory representation	Types	Methods	Model fit	Habitat-selection predictors
Avgar et al. [7]	Woodland caribou	Dynamics in animals’ habitat selection over landscape and time	Forage	Likelihood of animals moving with cognitive selection functions	Bayesian (MCMC)	1) Foraging qualities 2) Predator density (Wolf) 3) Moose habitat 4) Snow depth
Rheault et al. [86]	Mule deer	Differences between animals’ occurrences throughout time	Forage	SSF framework with an OD as a response variable in the model	Step Selection Functions (SSFs) with conditional logistic regression	1) Terrain ruggedness index 2) elevation 3) distance to treed edges 4) snow depth 5) land covers 6) NDVI 7) distance to well pads 8) distance to roads 9) previous ODs (past yr) 10) current ODs (prev 7 days)
Falcón-Cortés et al. [34]	Elk	Number of visits to the foraging path	Forage	A Markovian model with the probability of moving to patches	Bayesian (MCMC)	memory decay & memory use probability based on. 1) distance between patches 2) time since last visit to the patches
Thompson et al [102, 103]	Brown Bear	Current resource quality + time since the last visit	Forage	Hidden Markov Model with SSF Framework Likelihood of animals moving in non-stationary states with a cognitive map	Maximum-likelihood estimates (MLE)	1) distance from turbid water to riparian areas where bears selected 2) vegetation classes 3) density of ground squirrels and alpine sweet vetch 4) distance from human settlements
Oliveira-Santos et al. [76]	Feral hog	Utilization distribution of an animal’s trajectory	Home range	SSF framework with an OD as a response variable in the model	Step Selection Functions (SSFs) with conditional logistic regression	1) Land cover types 2) Time of day 3) BRB densities 4) Residence time
Northrup et al. [75]	Mule deer	Differences between animals’ occurrences throughout time	Home range	SSF framework with an OD as a response variable in the model	Hierarchical Bayesian regression (MCMC)	1) Tree 2) density of drilling well pads 3) average NDVI difference 4) major road density 5) terrain ruggedness 6) fat 7) age 8) natural gas facilities' density 9) total snow depth difference 10) pipeline density difference
Schlägel et al. [91]	Wolf	Time since the last visit	Territorial defense	Spatially explicit random walk model similar framework of. + selection-free movement kernel + selection functions	Maximum-likelihood estimates (MLE)	1) Prey density 2) Distance to territory boundary
Ranc et al. [85]	Roe deer	Food availability	Home range	Likelihood of animals moving with cognitive selection functions	Maximum-likelihood estimates (MLE)	1) Min. daily temperature 2) within state resource access 3) between-state resource access 4) illumination index (dawn & dusk) 5) changes in the illumination index
Ranc et al. [84]	Roe deer	Surrounding available habitats	Home range	Likelihood of animals moving with cognitive selection functions	Maximum-likelihood estimates (MLE)	1) Landcover - reforested 2) Landcover - agriculture 3) step lengths 4) Memory 5) Memory decay
Gurarie et al. [45]	Wolf	Predation success & Territorial marking	Territorial movement	A discrete choice modeling framework	Maximum-likelihood estimates (MLE)	1) selected zone per each trip 2) predation quality 3) boundary coverage 4) mass of prey item 5) time since predation events 6) number of kills 7) boundary sizes
Merkle et al. [67]	Mule deer	Differences between animals’ occurrences throughout time	Migration	SSF framework with a distance and turning angle bias as parameters in the model	Step Selection Functions (SSFs) with conditional logistic regression	1) Elevation 2) % tree cover 3) Distance to roads 4) Terrain position index 5) Integrated NDVI 6) Rate of Green-up 7) Distance to the previous route 8) Direction to the previous range

Popular analytical frameworks, such as Step-Selection Analyses (SSAs) [10, 35, 38, 104] have been used to identify signals of memory from observations of animal movements [67, 76, 84, 86, 90]. We focus our review on SSAs because of their flexibility and ease of use due to readily available statistical software [96], but also because of their continuous methodological development [56, 68, 81, 97]. Nonetheless, several additional challenges remain before these approaches can be widely adopted. These include technological challenges associated with managing tracking data and creating models with different “familiarity” or “memory” covariates. Here, we provide an overview of methods for parameterizing memory effects in SSAs to help guide practitioners wishing to identify or quantify the effects of memory on animal movement. We also offer several examples with annotated code and then discuss the strengths and limitations of current approaches and future directions for memory-informed movement research.

## Exploring how memory influences animal movements using SSAs

SSAs are widely used to quantify influences on animal movement [10, 35, 38, 104]. SSAs model movement in discrete time using two model components: (1) a selection-free movement kernel describing how animals move in the absence of habitat selection, defined using distributions of step length and turning angle, and (2) a selection function describing animal preferences concerning the habitat attributes at each step’s endpoint (Box 1). Model parameters in SSAs can be estimated using commonly available statistical software that implements conditional logistic regression [10, 35]. Because they allow one to model and predict dynamic space-use patterns using accessible and available software, SSAs are attractive to movement ecologists and are widely used to analyze animal tracking data [96]. Further, SSAs have strong connections to other popular methods for modeling animal movement, they have been shown to be equivalent to biased correlated random walks [22], and they can be approximated by diffusion-taxis models [83]. In addition, certain continuous time movement models can be recast as SSAs [31].

**Box 1:** Step-Selection Analyses (SSAs)

Step-Selection Analyses (SSAs) model the conditional probability, $p\left({s}_{t} | {H}_{t-1};{\beta }_{m,}{\beta }_{w}\right),$ of finding an individual at a location ${s}_{t}$ at the time *t* given a set of previously visited locations, ${H}_{t-1}$, using a selection-free movement kernel, $k\left({s}_{t} |{H}_{t-1};{\beta }_{m}\right),$ which describes how animals would move in the absence of habitat selection, and a movement-free habitat-selection function, $w\left({s}_{t};t,{\beta }_{w}\right),$ which describes the animals’ preferences for certain environmental features (e.g., variables representing resources, risks, and or other conditions; [62]):	
$$p({s}_{t}|{H}_{t-1};{\beta }_{m},{\beta }_{w}) =\frac{k({s}_{t}|{H}_{t-1};{\beta }_{m}) \cdot w({s}_{t};t,{\beta }_{w})}{\int_{{s}'\in U} k({s}'|{H}_{t-1};{\beta }_{m}) \cdot w({s}';{\beta }_{w}) ds'}$$	(1)
$${H}_{t-1} = {s}_{t-1},{s}_{t-2}, \ldots, {s}_{t-i}, \dots, {s}_{0}$$	(2)
${\beta }_{m}$ contains parameters in the step-length and turn angle distributions ${{(\beta }_{m1 },...,\beta }_{mq})$, and ${\beta }_{w}$ contains resource-selection parameters that quantify the attractiveness of different locations using a vector of selection coefficients (${{\beta }_{w1 },...,\beta }_{wp})$ for each environmental covariate ${{(r}_{1}({s}_{t}),...,r}_{p}({s}_{t}))$. ${s}{\prime}\in U$ describes all the locations within the spatial domain $U$. To calculate a step length, *sl*, two locations are required $,({s}_{t}, {s}_{t-1})$. Similarly, a turning angle, *ta*, is calculated using the current and the past two locations $({s}_{t}, {s}_{t-1},{ s}_{t-2})$.	

**Fig. 1:**
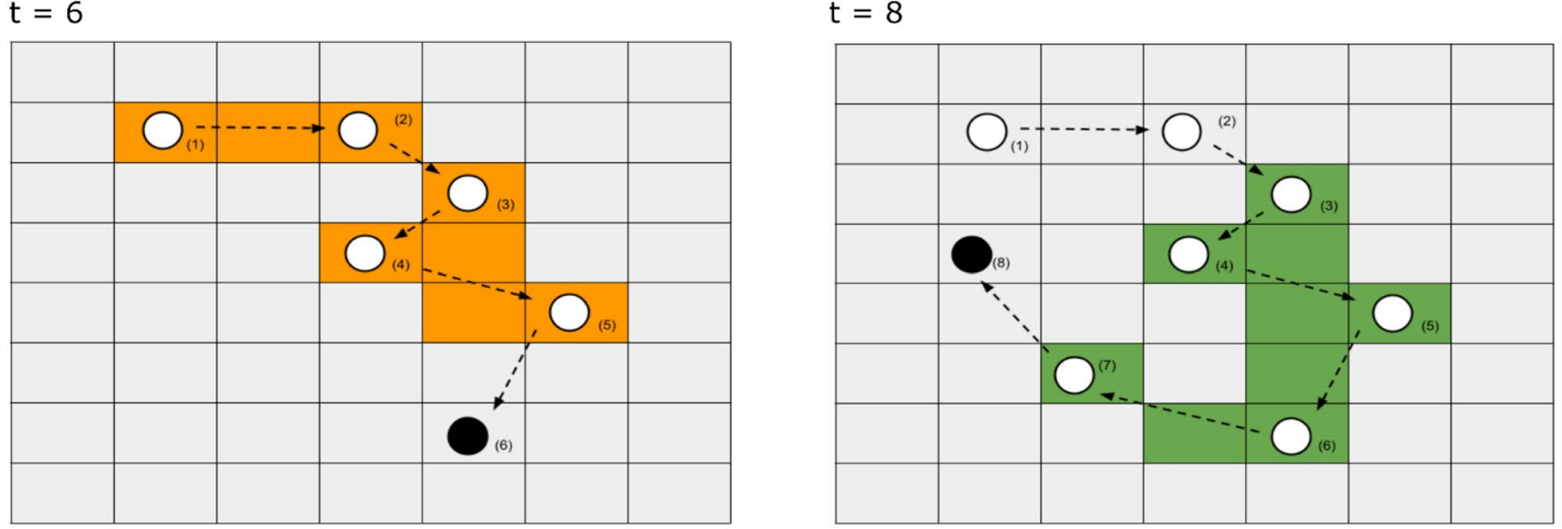
Spatial memory can be quantified using an animal’s occurrence distribution (OD) measured over some prior period (colored areas: 5-day periods at time 6 and time 8 = orange vs green) which captures an animal’s movement path and its uncertainty. A time-varying covariate can be constructed by updating the OD at regular time intervals. This updating step ensures that distant experiences are eventually forgotten and no longer play a role in driving animal movement. Users must choose an appropriate time window in the past for calculating the memory covariate and how often to update it

A variety of spatiotemporal familiarity covariates can be included in an SSA to model the effects of memory (Boxes 2, 3). For example, one may estimate an occurrence distribution (OD; [5, 36]) describing the relative use of the landscape over a specific period in the past (e.g., a continuous surface of either the relative intensity of use or a binary presence/absence variable). By including the OD as a spatial predictor in an SSA, one can evaluate whether an animal’s current landscape usage is biased toward or away from previously visited locations [76, 86, 117]. A notable limitation of this approach is the risk of overestimating the importance of familiarity due to unaccounted-for habitat attributes (e.g. if the OD reflects the distribution of an unobserved resource, see [79]). The OD approach further necessitates that users choose an appropriate time window in the past for calculating the OD (Fig. 1). Using a limited time window for calculating the familiarity covariate implies an abrupt memory decay function where past locations are memorized for a fixed amount of time and then forgotten completely. Alternatively, one could choose to continually update the OD from the first to the last location, which would imply the animal never forgets its past experiences. With either approach, the OD effectively weights all previously visited areas within the specified time window equally, regardless of how long ago the animal visited the location. Another option is to allow more recent (or distant) memories to have more influence on current movements by replacing the OD with a covariate representing the length of time since the animal last visited a location (TSLV). Or, one can create multiple ODs reflecting space use during the recent or the more distant past and allow the model to determine optimal weights given to short-term and long-term memories represented by these covariates (e.g., [76]).

**Box 2:** Guide for the memory-informed movement modeling within SSF framework

Question	Biological example	Method	Caveats, challenges, limitations	Implementation parameters or choices
Are animals more likely to visit areas that are more familiar to them?	Feral hogs primarily rely on recent experiences when making night time foraging decisions [76].	Occurrence Distribution (OD)	The effects of unmodeled environmental drivers may be wrongly attributed to memory and vice versa [79].	Time window for measuring past space use. Length of burn-in periods (how much early positional data to sacrifice to estimate the familiarity predictor?)
Do animals prefer to visit areas they have not visited recently? (and if so, is there an optimal return time based on foraging or predation experience?)	Predators like wolves and brown bears delay returning to previously visited kill sites so that prey numbers may rebound to normal levels [45, 92].	Time Since Last Visit (TSLV)	The effects of unmodeled environmental drivers may be wrongly attributed to memory and vice versa [79]. TSLV may be relatively homogeneous within an animal’s movement kernel.	“Patch” size (what is the typical spatial grain of the animal’s cognitive map?) Length of burn-in periods (how much early positional data to sacrifice to estimate the familiarity predictor?)
Do migratory animals use consistent migration routes to return to the same seasonal ranges each year?	Mule deer (*Odocoileus hemionus*) have been shown to use the exact same migration routes and seasonal ranges, likely relying on their memory of those spatial locations to help them navigate [67].	Distance and directional bias toward previously used areas across large spatial scales	The effects of unmodeled environmental drivers and social cues may be wrongly attributed to memory and vice versa [79].	Whether to use angular covariates or distance-to covariates. Time window for measuring past space use.

**Box 3:** Familiarity function in SSFs

Movement ecologists have described familiarity with different parts of the landscape that animals experienced using familiarity covariates, which we formalize via a familiarity function, *f* $.$ Including an exponential familiarity function allows the attractiveness of different locations to be governed by both environmental and familiarity covariates within the traditional SSF framework (${e}^{{\beta }_{w}r(s)}\cdot {e}^{{\beta }_{f}f\left(s\right)}={e}^{{\beta }_{w}r\left(s\right)+{\beta }_{f}f(s)}$). Examples of familiarity covariates include occurrence distributions (ODs) reflecting the intensity of past space use in the study area, time since last visit (TSLV), migratory distances between current and previously used paths, and angular covariates used to capture bias toward previously used migratory ranges:	
$$p({s}_{t}|{H}_{t-1};{\beta }_{m},{\beta }_{w},{\beta }_{f}) = \frac{k({s}_{t}|{H}_{t-1};{\beta }_{m}) \cdot w({s}_{t};t,{\beta }_{w}) \cdot f({s}_{t};t,{\beta }_{f})}{\sum_{{s}' \in U} k({s}'|{H}_{t-1};{\beta }_{m}) \cdot w({s}';t,{\beta }_{w}) \cdot f({s}';t,{\beta }_{f})}$$	(3)
$$f\left({s}_{t};t,{\beta }_{f}\right)=\{{e}^{{\beta }_{f} \cdot f({s}_{t})}\}$$	(4)

**Fig. 2:**
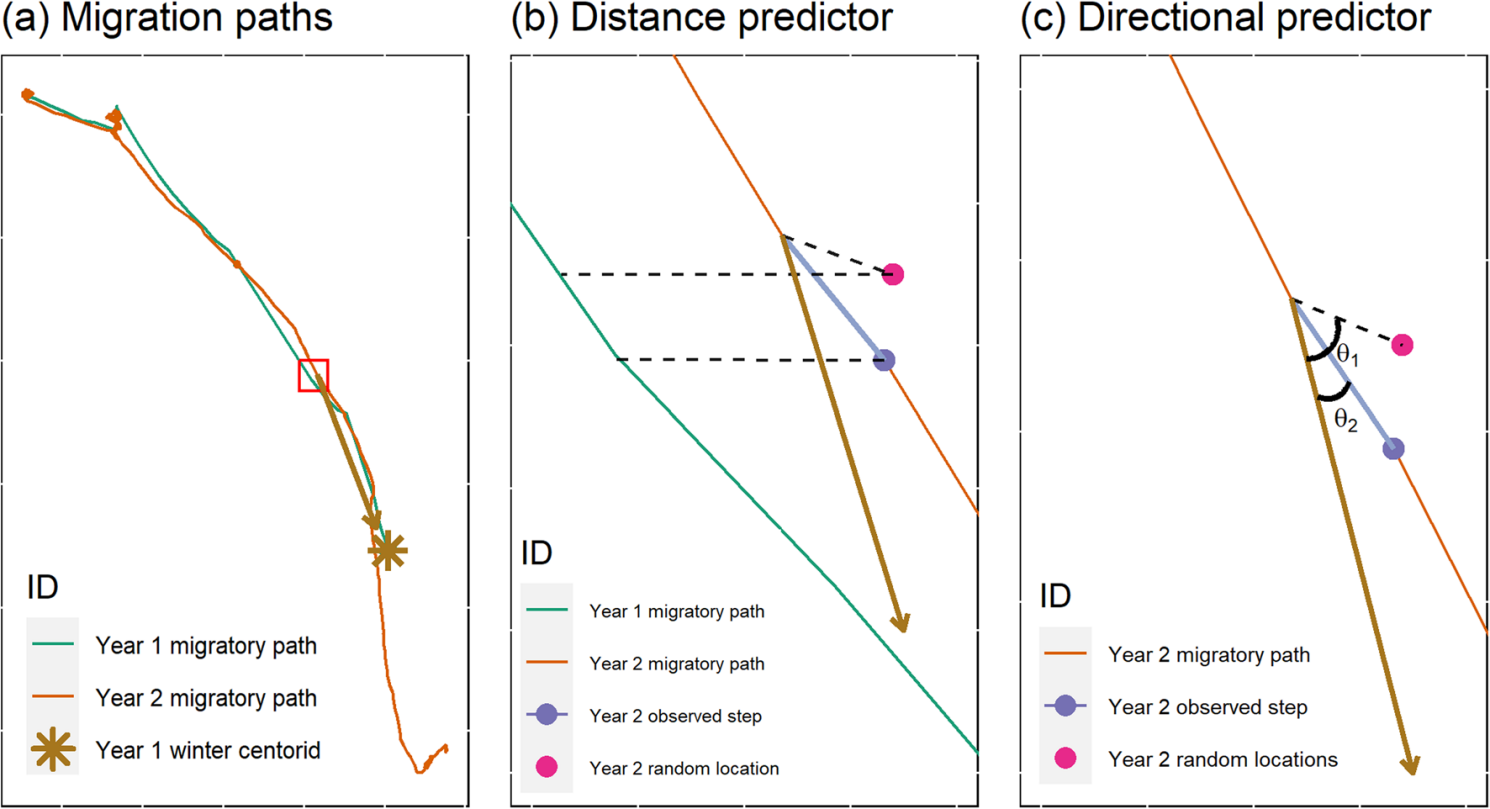
Familiarity covariates used in applications involving migratory animals. The red bounding box in panel (**a**) displays the area zoomed into for boxes b and c. Memory can be quantified using a distance predictor calculated as the minimum distance between current and past migratory paths (panel** b**). Specifically, we can calculate the distances (dashed lines) between Year 2 (observed [navy], random [pink]) locations and Year 1 (green) migratory paths. Memory can also be included as a directional bias predictor by comparing whether the current year’s steps are biased toward the previous season’s range (panel** c**). This bias predictor can be calculated using the angles, ${\theta }_{1, }{\theta }_{2}$, between the step (observed [navy], random [pink]) and the centroid of the previous year’s winter range

For migratory species that navigate relatively long distances, familiarity predictors could include distances between current and previous migratory trajectories or angular covariates that compare the direction of an animal’s movement in relation to a previous seasonal range (Fig. 2). These methods can describe an animal’s use of memory for navigation and capture its tendency to use familiar migration routes and consistent but seasonally varying home ranges [67].

Unless it is reasonable to assume that the animal lacked any memory at the onset of tracking, it is necessary to ‘sacrifice’ some early positional data to calculate the familiarity predictor. For some animals, including those that have long lifespans or live in highly seasonal environments, the ‘memory build-up’ period may need to be one year or more, unless there are reasons to believe memory (or its effect) decays over much shorter time scales. For example, Avgar et al. [7] used a full year of ‘memory build-up’ data to model woodland caribou (*Rangifer tarandus caribou*) space-use patterns over a subsequent year but have found no indication of memory decay within that time (indicating the need to use an even longer ‘memory build up’ period). Alternatively, studies of young or dispersing individuals and data from translocation experiments offer opportunities to model memory formation as individuals enter new and unfamiliar environments and learn how to navigate them efficiently [3, 15, 21, 32, 84, 85, 117] . However, datasets containing movements of individuals in unfamiliar landscapes are rare and difficult to obtain due to the high cost of translocation experiments and a tendency to avoid tracking juveniles due to often high mortality rates [93, 113].

## Case studies

In this section, we review memory-informed movement models for animal tracking data using 4 case studies. The first two examples, involving data from sandhill cranes (*Antigone canadensis*) and feral hogs (*Sus scrofa*), demonstrate how one can use a spatial familiarity predictor calculated from a past OD to explore whether animals retain information from their past experiences and for how long. The third example considers the migratory movements of mule deer and illustrates the use of two familiarity predictors formed using the minimum distance between the current and last year’s migratory paths and the cosine of the angle between the direction of a current movement step and the previous year’s centroid of locations. The last example, involving data from a brown bear, uses a spatiotemporal covariate to quantify how long it has been since the individual last visited spatial locations, and thus, how memory may influence revisitation rates. We provide a workflow to reproduce the main components of the habitat- and memory-based SSF from all the case studies, demonstrating the relative importance of memory-based and habitat metrics in an SSF of animal movement (see Appendix codes for the case studies). We also highlight challenges and decisions that the user must make when applying these methods to their tracking data (Box 2).

### Sandhill crane—‘fixed-time’ OD

Sandhill cranes breed throughout North America during the summer and migrate south for the winter. During their first year, juveniles migrate to overwintering areas with their parents and then disperse from the family group either during the spring migration or upon arrival to the natal territory the following spring [46, 100]. During the first few years of independence, subadult cranes typically make long-distance movements across the landscape during the summer; in contrast, the movements of breeding adults are largely constrained to their breeding territories [119]. Once cranes become successful breeding adults, typically between 4 and 6 years old, they use their accumulated knowledge of the landscape to return and nest in the same breeding area in subsequent years [74, 100].

**Fig. 3:**
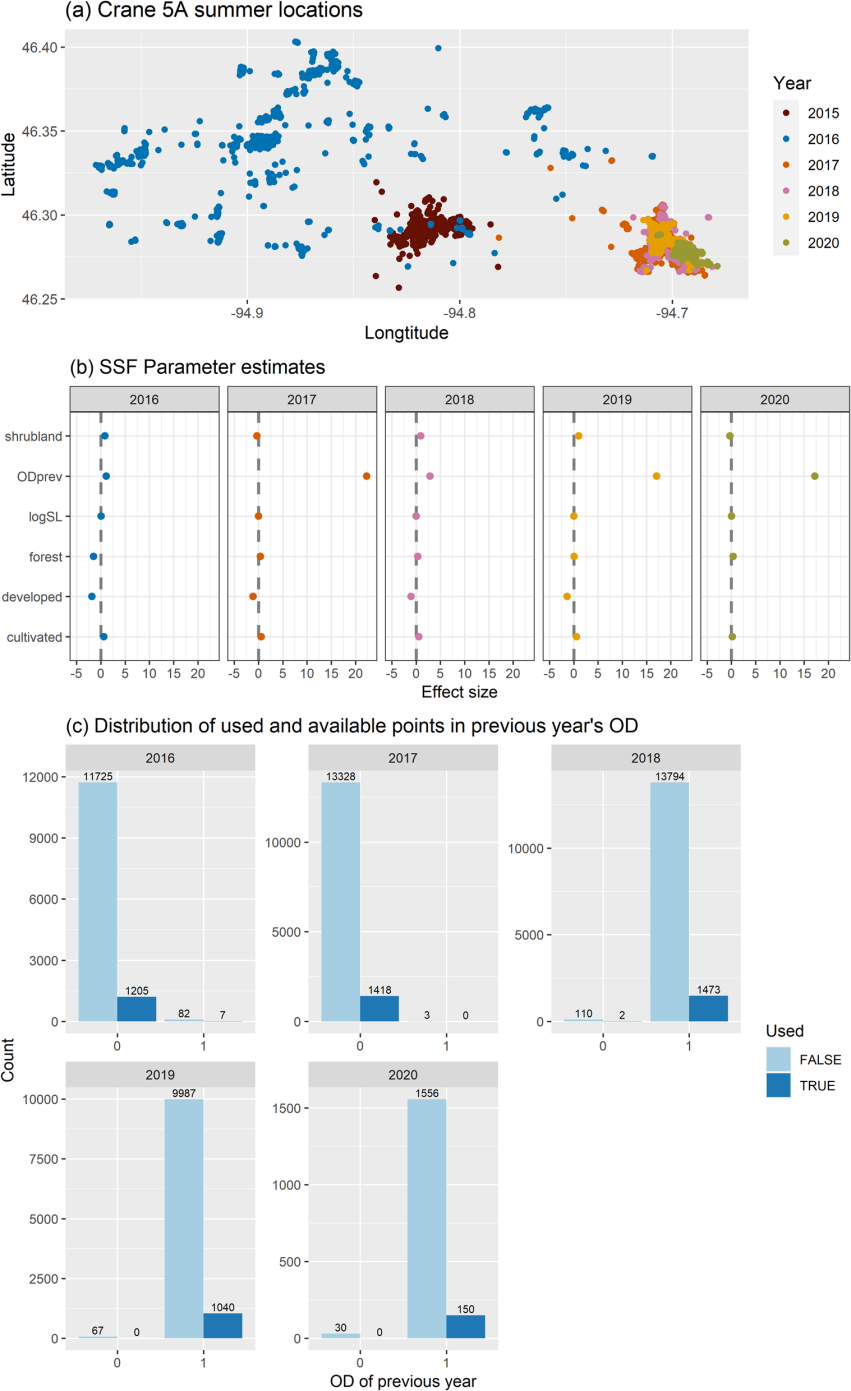
Visualization of Sandhill crane tracking data and coefficients from fitted step-selection functions with a memory covariate formed using an OD capturing previously visited locations. **a** Movement patterns of an individual sandhill crane during summer seasons (06/15-09/22) from 2015 to 2020. **b** Parameter estimates including those for the memory covariate (Odprev: OD from the previous year). **c** Distribution of each year’s used and available locations for sites (i.e., grid cells) that were (OD = 1) and were not (OD = 0) visited in the previous year. Numbers at the top of the bars indicate the number of locations in each group

As an illustrative example of how a sandhill crane’s space use constricts each year as the crane learns its landscape and develops a breeding territory, we consider a 5-year dataset of global positioning system (GPS) telemetry locations of a sandhill crane (with a 15-minute fix interval), starting from the time of fledging [118, 119]. A visualization of summer locations shows that the spatial coverage visited by the crane decreases each year as it ages, selecting locations it had previously visited (Fig. 3a). This pattern suggests that the crane may be using its past experience to decide where to establish a breeding territory.

To quantify the potential effects of familiarity on the crane’s summer movements, we calculated areas associated with the 95% contour of an OD estimated from the previous year’s summer locations using conditional probability density functions from a continuous-time movement model (where the conditioning ensures movements pass through observed locations; [36]). We then fit a separate SSF to each year of data, including the previous year’s OD as a predictor in the model. A positive coefficient associated with the previous year’s OD would suggest the crane prefers to revisit sites it visited in the previous year (compared to *equally accessible and otherwise identical sites* that it did not visit during the previous year, where accessibility is determined by the selection-free movement kernel; [35]). If the coefficient is negative, the opposite interpretation holds.

Coefficients associated with the previous year’s OD were positive in all years (Fig. 3b), even though the crane rarely revisited previously used locations in the first two years (Fig. 3c). Interpretation of the coefficients associated with categorical predictors can be difficult, as they reflect a ratio of ratios (use: availability ratio for one class versus the use: availability ratio of a reference class; [35]). The positive coefficients for the previous year’s OD in 2016 and 2017 reflect the fact that the use: availability ratio associated with grid cells visited in the previous year was higher than the use: availability ratio for grid cells that were not visited in the previous year. Thus, we end up with a positive coefficient for the OD predictor even though most of the areas encountered and used by the crane represent areas that were not visited in the prior year. Recall our interpretation of a positive coefficient, *if presented with two equally accessible locations* (same step length and turn angle required to reach both locations) that only differ in whether they had been visited in the previous year (i.e., they had the same landcover class), the crane would be more likely to select the location it was familiar with. A limitation of this model and application is that the crane was rarely presented with this type of choice. In the first two years, it was rarely found near sites it had previously visited whereas in years 3–5 it rarely visited areas it was unfamiliar with. This example highlights a limitation of the OD approach and the need for new methods that can capture tradeoffs in exploratory and informed movements as young individuals learn to navigate the landscape. Another limitation is that the birds may be selecting for an unobserved resource, one that we did not include in our analysis, which introduces a hidden correlation between their past and current space use.

### Feral Hog – OD with temporal variation: short- and long-term memories stratified by time of day

Hogs were introduced to the Pantanal wetland about 300 years ago, and currently represent the highest wild mammal biomass in this region. They are crepuscular-nocturnal, social, long-lived, cooperative animals that forage at the edges of water bodies and in ephemeral pools that become increasingly rare during the winter dry season. Hogs lack sweat glands and behaviorally mitigate heat stress by spending the hot hours of the day resting in forest patches.

As animals move, they can access and update their reference memory (long-term acquisition) and working memory (short-term acquisition) to navigate through space. Still, the stored spatial information may or may not be used depending on the current animal needs and context, which generates temporal heterogeneity (e.g., within the day, and seasons) in the use of that information. Oliveira-Santos et al. [76] modeled the hidden process underlying spatial memory acquisition using spatiotemporal covariates generated from Biased Random Bridge kernel density estimates based on residence time [13]. Specifically, for each individual step, they used previous locations to build multiple spatial maps, formed using different time windows, that could affect the hogs’ future movement decisions. These maps were then continuously updated as the individual moved through the landscape.

Oliveira-Santos et al. [76] considered 4 conceptually different hypotheses regarding how hogs process and use spatial information, combining long- and short-term memory with differential use of stored memory within the daily cycle. To represent long-term memory, familiarity covariates were constructed and constantly updated with all previous locations as the individual moved, whereas short-term memory was represented using a spatiotemporal covariate that kept track of just the last 3 days. Additionally, long- and short-term familiarity covariates were also built considering only daytime or only nighttime locations, which they referred to as long-term temporal and recent-temporal memory, respectively. When fitting models for these last two cases, movement steps taken at night or during the day were paired with familiarity covariates generated from previous locations collected only at night or day, respectively.

All tracked hogs strongly selected for previously visited areas, mainly those associated with short-term memory. Most of these individuals (65% of the tracked hogs) appeared to use working memory as part of their movement process, as covariates generated from recent nocturnal locations were better at predicting future nocturnal use than covariates generated from all time periods. Importantly, the effect of familiarity also varied within the day, being more important during the daylight hours when individuals were sleeping in well-known places than at night when animals were foraging and were more willing to take risks by walking through less familiar areas. Although hogs are acknowledged to have high cognitive skills and memory retention, Oliveira-Santos et al. [76] concluded that they relied mainly on recent spatial information because the distribution of prime food resources in the study area responds quickly to foraging pressure and changes in water levels.

### Mule Deer—migratory paths and angles

Many large ungulates are migratory, capitalizing on seasonal and spatial variation in food, predation, and hospitable conditions [9]. Mule deer are a concentrate forager (i.e., prefer to consume high-quality food) that display some of the longest terrestrial migrations in North America [51], spending winters in arid, low-elevation sagebrush, grassland, and desert ecosystems, and then migrating (up to 400 km) into montane ecosystems at higher elevations for summer.

Unlike some other migratory species (e.g., Sierra Nevada Bighorn Sheep; [14]), Mule deer migrate well outside their perceptual range (e.g., what they see, hear, and smell at a given moment), yet they display relatively strong fidelity to seasonal ranges [70] and migration routes [88]. On average, mule deer migrate on the same path during spring year after year 81% of the time [88]. In some cases, such migrations occur across relatively vast expanses of flat deserts, rolling hills, and thick forests, where sensory abilities such as vision may provide limited cues for navigation [89].

Evidence suggests that mule deer may navigate during migration by memorizing the path of their previous migration route and the general location of their seasonal ranges [67, 107]. The relative influence of these memorized spatial locations can be assessed in a movement model that first considers several other habitat features that may influence ungulate movement and space use. Merkle et al. [67] examined the relative role of memory usage versus local variation in habitat on mule deer navigation during migration. They found that variables indexing past experience (distance to the previous migration route and direction to the previous seasonal range) were 2–28 times more predictive of migratory movements than local variation in habitat. Alternative explanations for these long-distance migrations include following scent trails or other conspecifics; however, those explanations are not well supported due to the fact that these deer spend on average 81% of their migration walking on the exact same path as in the previous year [88].

### Brown Bear—time since last visit (TSLV)

Brown bears are opportunistic omnivores found in North America, Europe, and Asia [78]. Their life history strategies and dietary compositions vary greatly depending on the environment they live in [37, 41, 61], but their ability to navigate towards previously visited food patches is ubiquitous throughout their natural range [92, 102, 103, 116]. The “barren-ground grizzly bears” found in the Canadian Arctic are unique for many reasons, including their foraging and denning behavior [30, 64]. These bears’ food resources are only available for a short, albeit predictable, portion of their active seasons [30], so in addition to returning to the correct spatial location where food was present, bears must identify the temporal pattern of this resource and revisit the patch at the correct time. Ecologists interested in understanding how these bears incorporate memory into their movement patterns must implement models that account for these nonlinear temporal dynamics.

We modified the model developed by Schlägel and Lewis [90] so that it could be implemented in the step-selection framework and fit using conditional logistic regression. This required using distributions from the exponential family (gamma, von Mises) to model the distribution of step lengths and turn angles, respectively [10, 35]. In addition, we included both linear and quadratic terms to model the influence of time since last visit (TSLV) on the movement patterns of an adult female brown bear in the Mackenzie River Delta region of the Northwest Territories, Canada. This bear was immobilized and fitted with a GPS collar that recorded its location every 4h during the active season (the time in which the bear was out of its den).

**Fig. 4:**
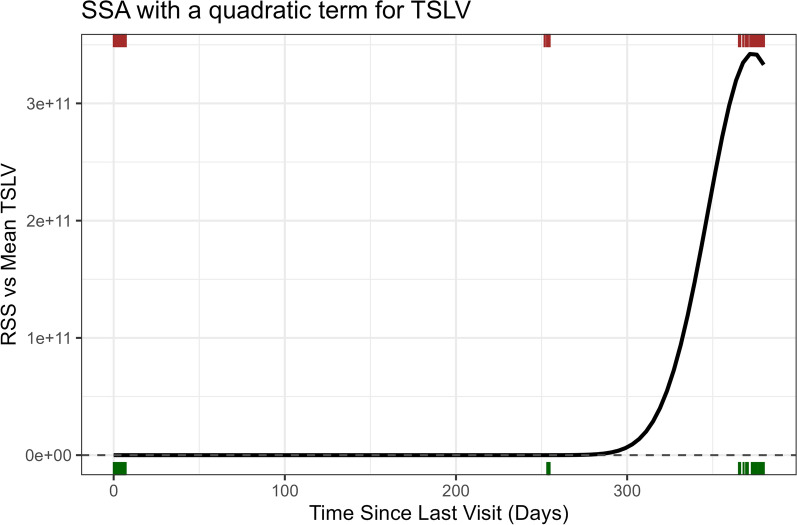
Visualization of the relative attractiveness of previously visited locations as a function of time since last visit (TSLV) modeled using linear and quadratic terms in a step-selection analysis of brown bear location data with the distribution of used (green, bottom) and available (brown, top) locations shown along the x-axis

We calculated TSLV as the difference between the current timestamp and the last time the bear visited a series of 2×2 km grid cells, updating the map at each observation time. This familiarity covariate allowed “revisitation” to occur when the animal was in the perceptual vicinity of an area it previously visited (i.e., within the same grid cell), without necessitating that the animal returns exactly to its previous coordinates [90]. We discarded the first year of location data as a “burn-in”, and set TSLV to 365 for any grid cells where TSLV was missing, effectively assuming that these cells had been visited just prior to the first observed location. The need for a burn-in period is a limitation of this approach but is necessary since we have no history of the bear’s past visits prior to the start of data collection. A sensitivity analysis can be performed to evaluate whether results change if the length of the burn-in period is increased or decreased. To analyze how selection strength varied with TSLV, we calculated the relative selection strength (RSS) at different TSLV values [10]. As with other habitat selection models, by including both linear and quadratic terms, we were able to identify a non-linear response to TSLV with intermediate TSLV values of approximately 350 days displaying the strongest selection (Fig. 4). These results agree with those of Thompson et al. [103], who also found that bears tended to revisit sites seasonally.

Although the TSLV approach can capture the potential memory effects of wildlife over time, the interpretation of the model may depend on the user’s choice of resource covariates. For example, we used berries as a seasonal resource covariate. However, the importance of the memory covariate (TSLV) might change if additional environmental covariates are added to the model [79]. Similar to Merkle et al. [67], Thompson et al. [103] compared their models with memory to “resource-only” models without memory, finding that the former models better fit the data. Yet, it is nearly impossible to perfectly quantify the distribution of food resources on the landscape, and “resource-only” models might have performed better if more environmental data could be acquired. Even when alternative models are considered and compared rigorously to a “memory model”, it is important to consider how these alternative models may fall short in quantifying their desired hypotheses.

## Discussion

Modern GPS tracking systems generate massive telemetry datasets by following individual animals over a long time on a global scale. With this abundance of available data, it is now possible to develop models that evaluate how memory relates to animal movement [73], which has inspired the recent development and application of many such models (see Supporting Information 1). Our review focused on approaches that account for familiarity with different areas of the landscape by including spatial, spatiotemporal, or angular covariates as predictors in step-selection analyses. These frameworks can be implemented using available statistical software for fitting conditional logistic regression models, which we demonstrate using multiple tracking data sets and annotated code examples. Although some data development is necessary before fitting the models (e.g., to create the memory predictors and generate random steps), users can leverage R packages to make these steps easier. For example, the ctmm package [19] can be used to calculate ODs, and the amt package [96] can be used to generate random steps necessary for parameterizing the model.

Individual-based models of animal movement are increasingly used to inform conservation and management at the population or even species levels [4, 48, 114]. Prime among these applications is the use of habitat-selection models to identify critical habitats, delineate range boundaries, and project spatial distribution across space or time [77]. A strong selection for certain habitats or environmental features and conditions may occur with or without memory, and it is important to consider how models that incorporate familiarity covariates may alter inferences. On the one hand, we might expect mild to moderate collinearity between familiarity covariates and other important environmental drivers, which can make it challenging to quantify their unique contributions [79]. On the other hand, accounting for familiarity and memory effects should reduce bias associated with estimators of habitat-selection strength. Consider an animal that is both attracted to certain habitats and to places it has used in the past; if we ignore the latter, we will consequently overestimate the former, putting more weight on habitat attraction than it truly has [79]. Similarly, it is important to consider the effect of environmental drivers when looking for influences of memory on animal movements [109]. Memory-free movement models that allow for habitat selection have been suggested as null models for evaluating evidence of memory [79]. An important caveat, however, is that it will be difficult in most cases to identify and measure all environmental features that animals select for. Thus, movement ecologists need to be cautious with attributing the effects of familiarity covariates to memory, even when the inclusion of familiarity covariates improves model fit relative to a null model containing known predictors of habitat quality. An additional benefit to considering memory effects in SSAs is the option to simulate these effects under various management scenarios [39]. For example, managers might be interested in assessing the likelihood of successfully relocating an animal and having it establish a home range in a new site [14, 85]. Explicitly modeling the process of building and responding to increasing knowledge of the landscape may be critical for obtaining reliable predictions.

Information gathering, an essential prerequisite for memory, is governed by sensory ecology [20, 27–29, 52], and it is in this context that we must also consider the limitations associated with the use of SSA to infer memory. The information available to the animal about any given spatial location (the ‘signal’) is a function of the animal’s position in relation to that location (the source of the signal), the strength of the signal (e.g., the intensity of odor, light, or sound), the overlap with similar signals coming from elsewhere on the landscape, and the animal’s sensory capacity to perceive and process the signal (which in itself may be a complex function of the animal’s morphology and physiology). Information can only be committed into memory if it is being perceived. Moreover, it is very likely that the weight given to memorized information (or its retention time) is modulated by the signal-to-noise ratio at which the information was perceived, and perhaps even by the desirability (‘good’ or ‘bad’) of the information (valence-dependent learning,[8]). The models we described here assume, for the most part, that animals sense and retain information from only ‘visited localities’, which are arbitrarily sized spatial units (typically corresponding to a single pixel of the available environmental data). Furthermore, most SSFs only assess what the animal can perceive within local ranges (i.e., what is nearby), though angular and distance-to-covariates allow modeling perception over larger spatial scales. Thus, SSFs are a useful, but extreme simplification of the true underlying sensory ecology.

More mechanistically inclined frameworks for explicitly modeling the sensory processes involved in memory buildup have been proposed (e.g., [7, 9, 65, 84, 102, 103]); however, fitting these models to wildlife tracking data is still a challenge. These frameworks typically include additional free parameters used to construct spatiotemporal covariates that determine how perceived habitat quality varies over time (e.g., by modeling how perception decays with distance from the individual, and memory decays temporally). As a result, it is not possible to fit these models using standard statistical software developed for SSAs. Instead, the parameters governing the spatiotemporal covariate must be estimated simultaneously with other habitat-selection and movement parameters through a custom-written likelihood function that can be optimized using Markov chain Monte Carlo or other numerical optimization methods. We include an example from Thompson et al. [103] in our supplementary material to demonstrate this approach. Methods that more realistically model the process of memory formation should be pursued, but we suspect that most practitioners will continue to explore the role of memory on animal movements using simpler models that can fit within the standard SSA framework using conditional logistic regression. The strength of this approach is that it can be easily and widely applied to tracking data. Still, these efforts should be complemented by additional experiments and more realistic mechanistic models to better understand the multifaceted ways that animals use memory to navigate their landscapes [84, 115].

## Supplementary Information

Supplementary Material 1.

## Data Availability

Data is provided within the supplementary information files and the datasets and code associated with the case studies can be found in a Github repository https://github.com/kimx3725/Memory_Movement/tree/main
