# Supplementary material for: Identifying signals of memory from observations of animal movements
Source: Mov Ecol. 2024 Nov 18;12:72. doi: 10.1186/s40462-024-00510-9 (PMC11575436; doi:10.1186/s40462-024-00510-9)
Supplement: Supplementary file 1 — Supplementary Material 1. [file 40462_2024_510_MOESM1_ESM.zip › Memory_Movement-main/Brown_Bear_Thompson_etal_2022/code/final_script.html]

final\_script


# final\_script

#### Peter R. Thompson

#### 2022-09-29

This script will conduct all the analyses necessary to replicate the analyses conducted by Thompson et al. (2021) for one individual bear in the population. This individual (ID: GM1046) was chosen because it had more data than most of the population, having been collared for four conseucitve years. Its movement track is depicted in Figure 3 of Thompson et al. (2022). The steps reuqired to complete this analysis, given the necessary input data, are detailed below. This includes loading some necessary R packages and defining a number of fairly complex functions. See the comments below for further detail.

```
library(TMB) # Template Model Builder (TMB) is the primary engine we use to fit the very complex models. It employs automatic differentiation techniques to much more accurately approximate a likelihood function, which greatly aids in the speed and precision of numerical optimization. When using TMB, the likelihood functions are written in C++; the necessary files are included in the repository here.
library(circular) # necessary for loading in the von Mises distribution, our angular distribution of choice for characterizing turning angles
```

I’ve written a few (fairly large) functions that help us run our analysis in a streamlined fashion. These functions are omitted from the “knitted” version of this file but can be viewed in the source code (.Rmd) version.

Now that we have read in all our functions, we can conduct our analysis, displayed below. Before running this line you’ll need to set your working directory to a folder that contains the folder “GM1046\_input” as well as all the necessary C++ files.

```
# First we read in our data and get it in the proper format for analysis.
data_raw = read_SSF_raw_data("GF1143_input")

# In this function, we fit all four models at once to our input data. See the above code block for further detail on what this function does (in short, it uses TMB to fit the SSF models to the data).
fit = fit_SSF(data = data_raw, init_cond = 3, lb_sdtau = 18, BIC = TRUE,  mu_stationary = 30, control = list(eval.max = 1000, iter.max = 1000),
              models = c('null', 'resource', 'memory', 'combination'))

# This allows us to store our results as a CSV file for further analysis
write.csv(fit, "model_fits_1143.csv")
```

Now we can analyze our results. What do we see? Let’s first use BIC to identify which model produced the most parsimonious explanation of the patterns observed in this bear’s movements.

```
fit[c(1, 5, 16, 30), c("model", "IC")]
```

```
##                      model       IC
## steplength            null 3455.413
## 1                 resource 3446.074
## 21             memory-only 3448.229
## 82         resource-memory 3435.895
```

We can see that here, the resource-memory model produced the lowest BIC. Let’s look more at the parameter estimates for this model.

```
fit[fit$model == "resource-memory", c("parname", "estimate", "CL", "CU")]
```

```
##     parname      estimate            CL            CU
## 16   rho_ns  3.874247e-01  3.239683e-01  4.508812e-01
## 22    kappa  7.219014e-01  4.677797e-01  9.760231e-01
## 32   beta_0  1.158694e-01  1.879484e-06  9.998906e-01
## 42    beta1  1.474086e-01 -2.097324e-02  3.157904e-01
## 52    beta2 -7.422739e-04 -1.243211e-03 -2.413363e-04
## 62    beta3  4.982796e+00  3.289957e+00  6.675634e+00
## 72    beta4 -3.462176e-02 -6.443880e-02 -4.804719e-03
## 82    beta5  3.680359e-05 -9.715155e-05  1.707587e-04
## 91    beta6 -2.736287e-05 -1.433327e-04  8.860692e-05
## 101  beta_d  9.996608e-01  3.293133e-02  1.000000e+00
## 111      mu  3.522245e+02  3.459817e+02  3.584673e+02
## 12    sigma  1.453948e+01  1.529847e+00  2.754912e+01
## 13   lambda  1.787071e-01  7.140557e-02  3.810800e-01
## 14    gamma  8.479306e-01  7.744063e-01  9.005689e-01
## 15    alpha -1.189024e-01           NaN           NaN
```

Note that some of the confidence intervals may disagree with what was shown in Thompson et al. (2022). This is because there, we used likelihood profiling to approximate the confidence intervals, rather than the output given by a simple call to the R optimizer.
