# Supplementary figures and images for: Identifying signals of memory from observations of animal movements
Source: Mov Ecol. 2024 Nov 18;12:72. doi: 10.1186/s40462-024-00510-9 (PMC11575436; doi:10.1186/s40462-024-00510-9)

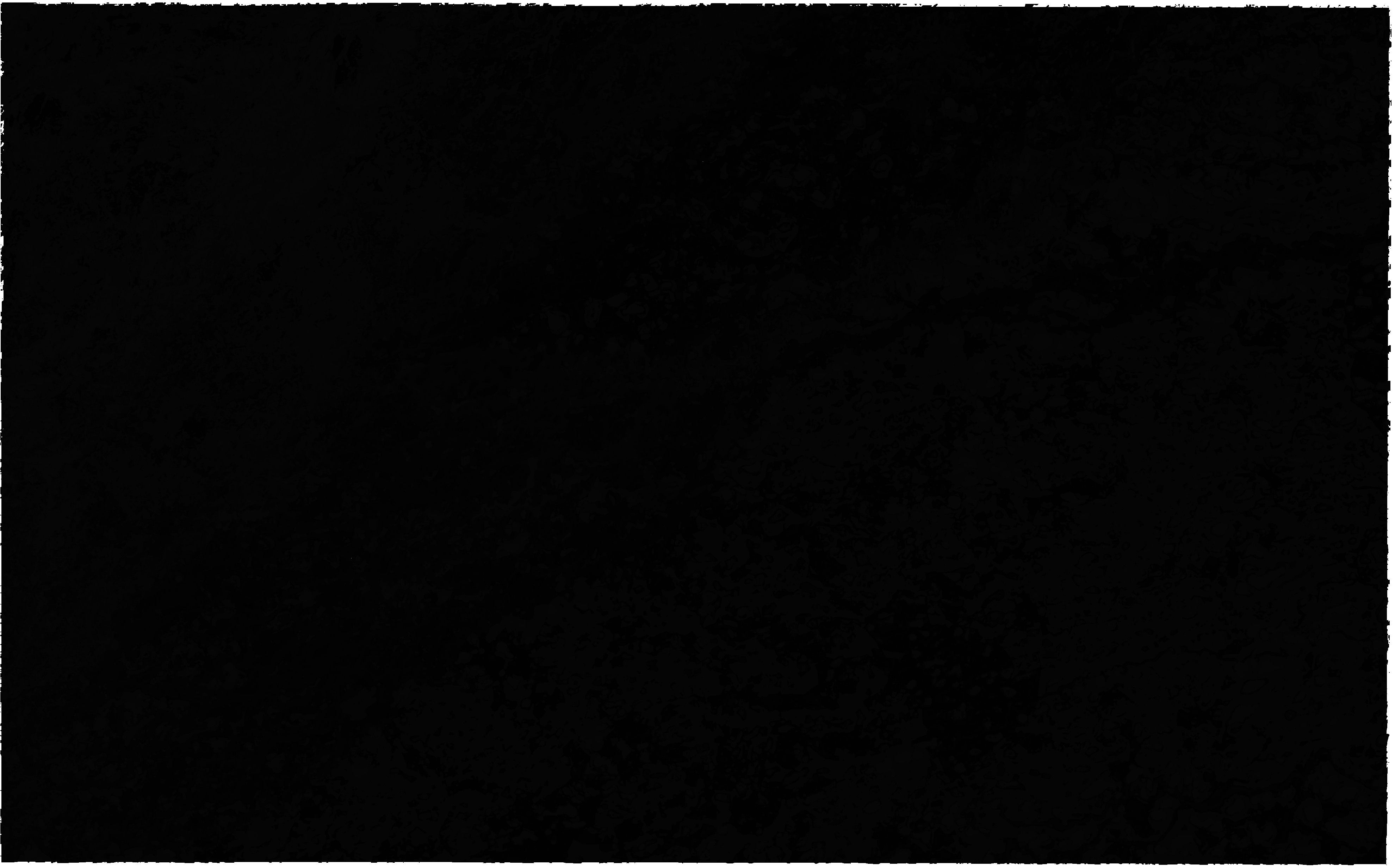

Supplement: Supplementary file 1 — Supplementary Material 1. [file 40462_2024_510_MOESM1_ESM.zip › Memory_Movement-main/Feral_Hog-Oliveira-Santos_etal_2016/data/UTM.tif]

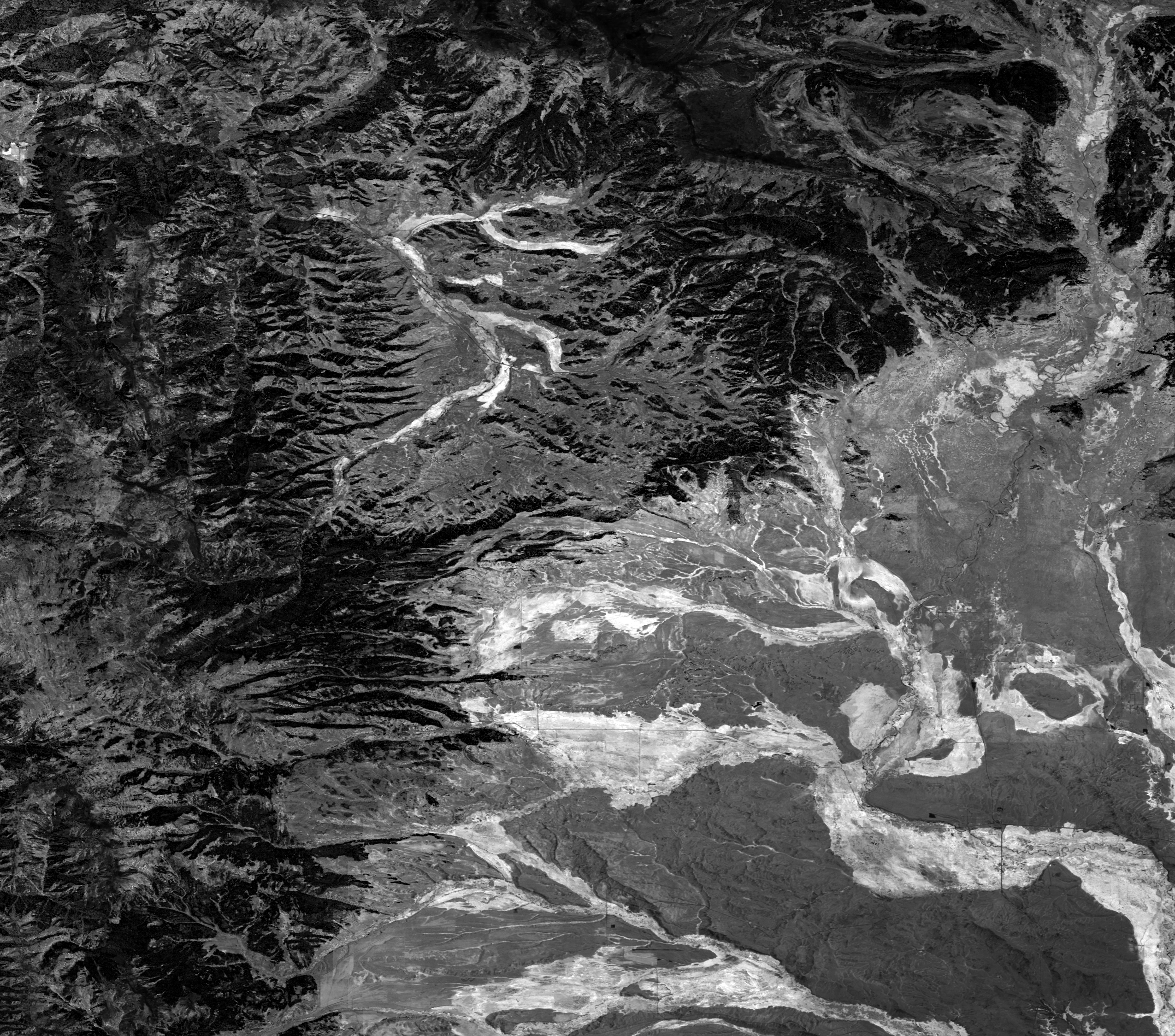

Supplement: Supplementary file 1 — Supplementary Material 1. [file 40462_2024_510_MOESM1_ESM.zip › Memory_Movement-main/Mule_Deer-Merkle_etal_2019/data/RAP_2015_Biomass_ForbsGrasses.tif]

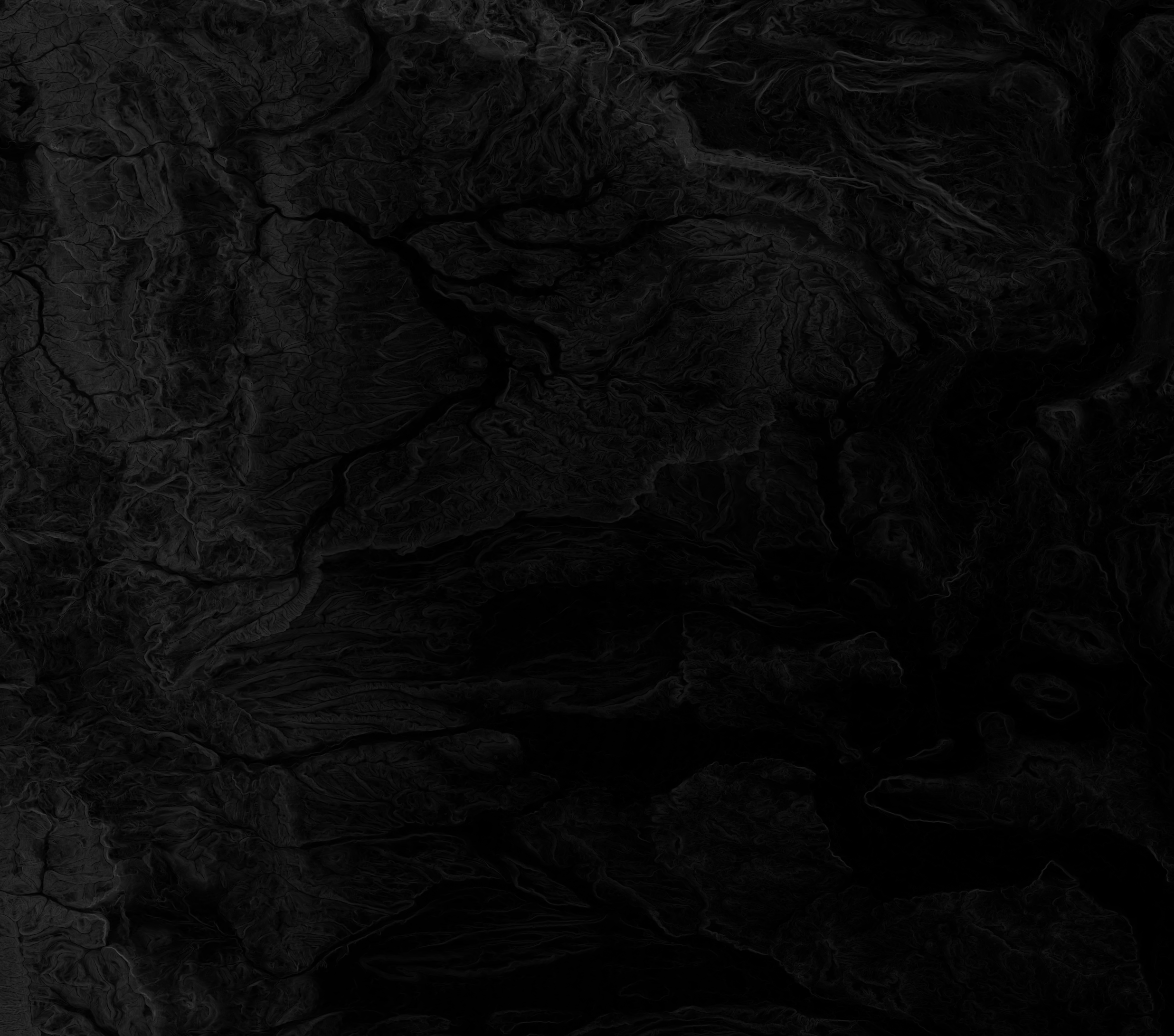

Supplement: Supplementary file 1 — Supplementary Material 1. [file 40462_2024_510_MOESM1_ESM.zip › Memory_Movement-main/Mule_Deer-Merkle_etal_2019/data/Slope30m.tif]

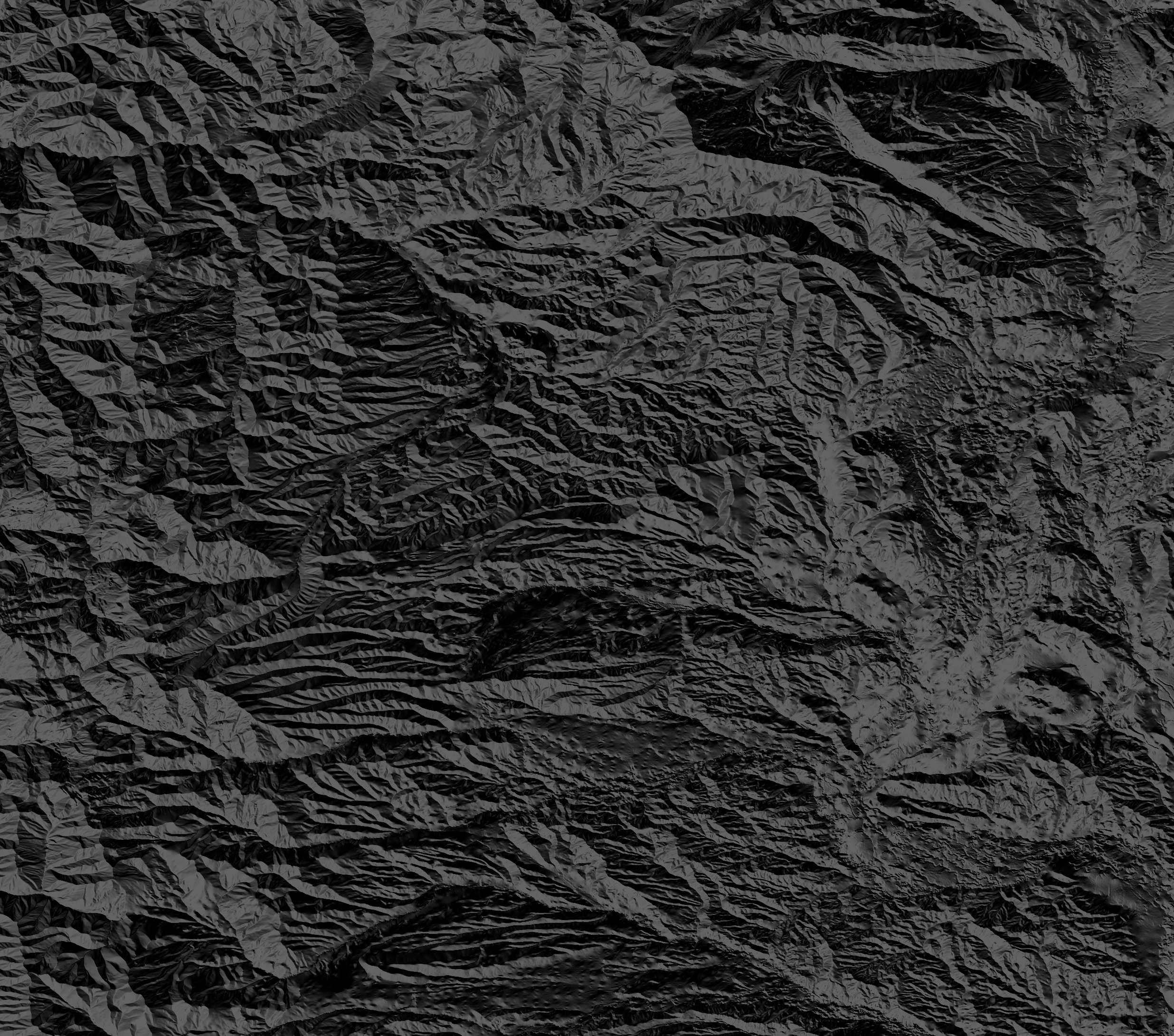

Supplement: Supplementary file 1 — Supplementary Material 1. [file 40462_2024_510_MOESM1_ESM.zip › Memory_Movement-main/Mule_Deer-Merkle_etal_2019/data/TRASP30m.tif]
